# Chronic Kidney Disease and Periodontitis Interplay—A Narrative Review

**DOI:** 10.3390/ijerph20021298

**Published:** 2023-01-11

**Authors:** Sorana Florica Baciu, Anca-Ștefania Mesaroș, Ina Maria Kacso

**Affiliations:** 1 Department of Dental Propaedeutics and Esthetics, Faculty of Dentistry, “Iuliu Hațieganu” University of Medicine and Pharmacy, 32 Clinicilor Street, 400006 Cluj-Napoca, Romania; 2 Department of Nephrology, “Iuliu Hațieganu” University of Medicine and Pharmacy, 2 Babes Street, 400012 Cluj-Napoca, Romania

**Keywords:** chronic kidney disease, periodontitis, oral microbiota, pathophysiologic mechanisms, treatment

## Abstract

Periodontitis (PO), a chronic microbially-induced inflammation of the supporting tissues of the tooth, is linked to various systemic diseases. We analyze its bidirectional relationship to chronic kidney disease (CKD), a major health-care problem with impressive excess mortality. Overwhelming associative relationship between CKD and PO are analyzed. Major pathophysiologic mechanisms that link CKD to PO are then presented: systemic inflammation, endothelial dysfunction, and imbalance of oxidative stress characteristic of CKD have a role in PO development and might influence escape mechanisms of oral microbiota. Subclinical local and systemic inflammation induced by PO might influence in turn CKD outcomes. Homeostatic changes induced by CKD such as mineral bone disorders, acidosis, uremic milieu, or poor salivary flow are also relevant for the occurrence of PO. There is insufficient evidence to recommend a standardized diagnostic and therapeutic approach regarding association of PO to CKD.

## 1. Introduction

Chronic kidney disease (CKD) is a major socio-economic and health-care problem, with a global prevalence of almost 7 billion people worldwide [1]. Globally, 8724 out of 100,000 people have CKD, a number exceeding that of diabetes by 80% and ten times that of cancer patients [2] and increasing; it is predicted that, by 2040, CKD will be the 5th cause of death worldwide [1].

The leading cause of CKD is diabetes, followed by hypertension and glomerular diseases [3], but irrespective of the initial nephropathy, once a critical mass of nephrons has been lost, kidney disease is generally progressive towards more advanced stages, characterized by increased morbidity and mortality. The burden of CKD is derived both from management of end-stage kidney disease and from impact of complications of CKD and important comorbidities. The absolute risk for death increased exponentially, with decreasing renal function being almost double the general populations in advanced CKD [4]. Mortality excess in CKD patients is impressive, and the main cause of mortality is cardiovascular problems [5]. However, increased cardiovascular risk and mortality is also present in pre-dialysis stages, with CKD being a major cardiovascular risk factor [4]. Mortality, morbidity, and impaired quality of life in CKD patients result in significant decrease in disease-adjusted life years associated with this condition [6]. There are efficient ways to decrease progression of CKD and improve outcomes of these patients. The efficacy of these therapies, including blockade of the renin-angiotensin system and sodium glucose transporter 2 inhibitors, is markedly increased when applied early in CKD. Therefore, screening patients at risk for CKD and timely diagnoses is a healthcare priority [7].

Traditional cardiovascular risk factors, such as those identified in the Framingham hearth study [8], fail to accurately predict outcomes in CKD patients. Therefore, research has focused on “non-traditional” risk factors such as systemic inflammation or oxidative stress [9], which might be influenced by periodontitis (PO).

PO is a highly prevalent disease, affecting around 50% of the population [10]. Investigations on trends of prevalence based on the estimates from the Global Burden of Disease study found that in 2019, there were 1.1 billion prevalent cases of severe PO worldwide with an 99% increase globally over the past three decades (1990–2019), mostly in limited-resource countries; population growth, aging, and changes in age-specific rate accounted for 67.9%, 18.9%, and 12.2% of this growth, respectively [11].

PO is a chronic microbially-induced inflammation of the supporting tissues of the tooth that results in loss of periodontal attachment, alveolar bone, and finally of the tooth. The homeostasis of the periodontal tissues is ensured between the hosts’ immune-inflammatory reactivity and the subgingival microbiome [12]. Factors that can influence this symbiosis between the hosts’ immune-inflammatory reactivity and the subgingival microbiome are excessive bacterial load in the subgingival areas and an improper, usually excessive, immune response of the host induced by genetic, behavioral, or systemic factors [13], with recruitment of immune cells and aberrant, dysregulated synthesis of proinflammatory cytokines, chemokines, metalloproteinases, and activation of osteoclastic activity [14]. Psychological discomfort, stress, and problems in interpersonal relations caused by PO impacted on the quality of life of the patients [15].

POs’ severity is influenced by various pathological conditions and, in turn, PO may also impact systemic health, and it has been independently associated with many chronic noncommunicable diseases. The evidence supporting these associations mainly focused on diabetes, pregnancy, and cardiovascular disease, but other conditions such as obesity, auto-immune conditions, certain cancers, respiratory diseases, and cognitive disorders including Alzheimer’s disease must be considered [16,17,18]. Less is known about the link of PO to CKD.

This review aims to present associative relationships between these two conditions and report concepts on the major pathophysiologic mechanisms that link CKD to PO, such as particularities of oral microbiota, inflammation driven by innate and adaptative immunity, endothelial dysfunction, and imbalance of oxidative stress. Lastly, we will discuss the treatment of periodontal disease in CKD patients.

## 2. Epidemiology of CKD and PO

### 2.1. Association of PO and CKD

Prevalence of PO in CKD: PO is more common in pre-dialysis CKD patients than in the general population, and this has been consistently described in various cohorts [19,20,21,22,23,24]. Ethnic differences may, however, play a role: prevalence of PO in CKD patients was more important in non-Hispanic Blacks and Mexican Americans, but not so in non-Hispanic Whites in the National Health and Nutrition Examination Survey (NHANES) cohort [24]. As CKD progresses, the prevalence of PO increases, being more important in advanced stages [4,6] when compared to earlier stages of CKD [25,26]. In dialysis patients, the prevalence of PO exceeds 50% of patients [27,28,29,30], and in some reports approaches 100% in peritoneal [31] and hemo-dialysis (HD) patients [32].

*Prevalence of CKD in PO patients:* Conversely, kidney disease, defined as decreased glomerular filtration rate (GFR), hematuria, or albuminuria, was more frequently reported in patients with PO in different cohorts [33,34]. These associations between PO and CKD are confirmed in several systematic reviews and metanalysis [35,36,37,38,39]. In the most recent one, by Serni et al., PO was identified as a frequent CKD comorbidity, ranging from 34.35 to 93.65% in the different studies included, with a higher prevalence in advanced CKD [19].

*Link of severity of PO and CKD:* There seems to be a link between severity of PO and that of CKD: PO is more severe in CKD than in the general population [25], subjects with more severe PO are more prone to having increased severity of CKD [20,40], and length of renal replacement therapy is associated with severity of PO in both HD [41,42] and peritoneal dialysis patients [31,43].

### 2.2. Potential Causality and Biderctional Determinism

From cross-sectional studies, causality cannot be inferred; indeed, some overlap of CKD and PO might be expected, as they are both very common conditions, and prevalence increases with age. However, there is mounting evidence that CKD and PO might have common pathophysiologic mechanisms, based on increased inflammatory state, impaired immune clearance, and other factors such as xerostomia, gingival hypertrophy, or uremic milieu. PO was predictive for occurrence of CKD in large epidemiologic studies such as NHANES III (National Health and Nutrition Examination Survey) [22] or other prospective cohorts [44,45,46]. PO is identified as a non-traditional risk factor for GFR decline [47]. Vice-versa, CKD is also associated with progression of periodontal disease in longitudinal studies [48].

Complex statistical analysis revealed that the association of PO with CKD has most likely bidirectional determinism. As such, in a recent analysis data from a prospective study (RIISC) (Renal Impairment in Secondary Care) with longitudinal data on PO, kidney function, and oxidative stress available, structural equation modelling was used to test causal assumptions between PO and kidney function. This study was positive in that 10% increase in periodontal inflammation resulted in a 3.0% decrease in renal function and a 10% decrease in renal function resulted in a 25% increase in PO, and the common denominator is thought to be oxidative stress [49].

Genetic predisposition does not seem to be an important pathogenic factor between PO and CKD: using genome-wide association studies, single nucleotide polymorphisms (SNP) identified as genetic causes of PO were not related to kidney function and vice versa, and multiple SNP identified as genetic causes of CKD were not associated with PO, using 2 large genetic databases of PO (Gene-Lifestyle Interactions in Dental Endpoints—GLIDE Consortium) and of CKD (Chronic Kidney Disease Genetics—CKDGen Consortium) [50].

### 2.3. PO Increases Morbidity and Mortality in CKD

The main cause for the increase in mortality of patients with both CKD and PO is the renal condition. As a result, PO can be studied as a modifier of this risk. Not only is there an epidemiologic link between CKD and PO, but the presence of periodontal disease was found to exacerbate cardiovascular morbidity [51] and the risk of mortality in prospective observational studies in HD [52,53] or pre-dialysis patients [54]. Similar data are also derived from large prospective cohorts like the ARIC (Atherosclerosis Risk in Communities) study [55] or NHANES III [56]. A meta-analysis confirmed that PO significantly increases the risk of all-cause mortality in CKD [57].

However, negative studies are also reported: a recent large database study from Taiwan does not support the idea that PO increases mortality in CKD patients or that it influences progression of kidney disease [58], and in the study of Palmer et al., moderate to severe PO in HD patients was associated with a lower risk of all-cause and cardiovascular mortality than those with no or mild PO when controlling for potentially confounding variables [59].

### 2.4. Need for Uniform Definitions

Controversial findings in different studies are at least in part due to disparate sets of criteria that have been used to diagnose PO and CKD. Currently, according to KDIGO (Kidney Disease Improving Global Outcomes) [60], CKD is defined as abnormalities of kidney structure or function, present for more than 3 months, with implications for health. These consist of decreased GFR < 60 mL/min/1.73 m^2^ or the presence of one of more markers of kidney damage, such as albuminuria, urine sediment abnormalities, electrolyte, and other abnormalities due to tubular disorders, abnormalities detected by histology, structural abnormalities detected by imaging, and history of kidney transplantation. The staging of CKD relies on a decrease of estimated GFR and includes 5 categories: G1 (GFR > 90 mL/min/1.73 m^2^), G2 (GFR 60–89 mL/min/1.73 m^2^), G3a (GFR 45–59 mL/min/1.73 m^2^), G3b (GFR 30–44 mL/min/1.73 m^2^), G4 (GFR 15–29 mL/min/1.73 m^2^), and G5 (GFR < 15 mL/min/1.73 m^2^). Three categories for albuminuria are also defined based on urinary albumin to creatinine ratio or 24 h urinary albumin excretion: A1 (<30 mg/24 or mg/g creatinine), A2 (<30–300 mg/24 or mg/g creatinine), and A3 (>300 mg/24 or mg/g creatinine). The combination of the G and A categories defines severity of CKD, risk of progression, risk of cardiovascular morbidity and mortality, and need for intervention. This definition, dating back to 2012, is globally implemented. Nevertheless, across studies that addressed the link of PO to CKD GFR, estimation equations used are not uniform and GFR thresholds used for inclusion of patients are widely different, with many studies including only dialysis patients.

Periodontal diseases represent a wide range of inflammatory conditions that affect the supporting structures of the teeth (the gingiva, bone, and periodontal ligament), which could lead to tooth loss and contribute to systemic inflammation [61]. The currently accepted definition of PO was recently provided by a 2018 classification scheme of periodontal and peri-implant diseases and conditions established by the European Federation of Periodontology/American Academy of Periodontology (2018 EFP/AAP) [62]. This uses complex staging and grading systems with the current PO case definition based on clinical attachment loss (CAL) severity and number of affected sites; staging based on the severity and the extension of PO at presentation as well as on the complexity of the required treatment and grading based on biological features of the disease, the former rate of periodontal destruction and the risk factors anticipating further PO progression, poor outcomes of therapy, and the general health impact [63,64]. A new definition has just recently been validated [65], and should be used in future efforts to strengthen global surveillance of PO, having in view the local and general disastrous impact of this disease. Inconsistency in definitions used for both conditions significantly accounts for the controversial findings in different studies.

## 3. Pathogenic Link of PO and CKD

### 3.1. Oral Microbiota: Dysbiosis in PO and CKD

The human microbiome consists of the totality of microorganisms in the human body, which are distributed in various organs, with the oral cavity being an important site. Changes in the composition of microbiome or in its metabolites can trigger and aggravate several systemic diseases [66]. The human microbiome is highly personalized, and its current understanding has tremendously improved with advancement of genome sequencing techniques. In PO, the normal microbiome is altered in favor of periodontopathic bacteria from the subgingival microbiota, including Aggregatibacter actinomycetemcomitans, Fusobacterium nucleatum, Tannerella forsythia, Porphyromonas gingivalis, Prevotella intermedia, and Treponema denticola [67], which cause direct and indirect bacterial-induced periodontal destruction—the so-called “red complex” pathogenesis model [67]. Keystone pathogens are species with disproportionately large effects on their communities relative to their abundance [68], being able to induce protracted inflammation.

Alterations in systemic or local homeostasis triggered by disease in distant organs could be a major factor in altering oral microbiota. As such, the interrelation of CKD and gut microbiome is well-documented, but changes of oral microflora in CKD are less studied [69]. Oral keystone bacteria associated with PO such as Porphyromonas gingivalis, Tannerella forsythia, Treponema denticola [70], Aggregatibacter actinomycetemcomitans, and Actinomyces dentalis [71,72,73] have been found to be more prevalent in CKD patients than in the general population.

Antibodies to selected periodontal pathogens, including Porphyromonas gingivalis, are associated with increased odds of having an impaired renal function [74].

### 3.2. TLR-Mediated Activation and Microbial Escape Mechanisms in PO and CKD

Alterations in oral microflora can influence distant organs in various ways, such as translocation and bacteremia or toxic effects of bacterial compounds [75], but the most important mechanism seems to be mediated by the immune system and inflammation. A putative example for the role of oral microorganisms in renal disease is poststreptococcal glomerulonephritis. However, a more subtle, persistent, and relevant crosstalk between oral microflora and immune system of CKD patients might be an important factor in promoting systemic inflammation. The “red zone” bacteria of PO can escape immune response in various ways, leading to protracted inflammation which can affect multiple organs, including the kidneys. In particular, Porphyromonas gingivalis keystone pathogen of the PO-provoking subgingival microbiota impairs local immune system through its capacities to evade and impair elements of the host immune-inflammatory system, which alters the growth and development of the entire subgingival biofilm. Lipopolysaccharide (LPS)-mediated TLR activation through My88 (myeloid differentiation primary response gene 88 (MyD88) normally results in nuclear factor k beta (NF-kB)-mediated transcription of proinflammatory cytokines, which are meant to recruit inflammatory cells of the adaptative immunity and limit or halt inflammation. However, alternative intracellular cascades such as phosphatidyl inositol 3 kinase (PI3K) or complement factor 5 (C5)-mediated cyclic AMP (cAMP) induction [76], and perhaps other processes that block normal phagolysosome activation of macrophages and neutrophils, cause macrophage immunosuppression and enhanced pathogen survival in vitro and in vivo [76]. Consequently, bacteria escape clearance from the immune system but contribute to protracted, inefficient inflammation, and the normally homeostatic host–microbial interactions are changed toward destructive relationships.

LPS-mediated activation of the innate immune system might also have systemic consequences relevant for distant organs, such as the kidney. In fact, endothelial cells express TLR2 and TLR4 in diabetic environment, including in renal microcirculation and translocated Porphyromonas gingivalis from the subgingival biofilm, bind to these cell surface receptors, activating endothelial cells and causing overexpression of adhesion molecules like VCAM1 and E-selectines. This, in turn, leads to leukocyte margination and glomerular, as well as tubulointerstitial inflammation [77].

On the other hand, CKD-induced alterations of microenvironment also play a permissive role: both innate and adaptive immunity are impaired. In CKD, antigen presentation capabilities of dendritic cells and macrophages are diminished [2], leading to decreased efficiency in monocyte stimulation, impaired phagocytic capabilities of neutrophils, and diminished cytokine secretion [78]. Inefficiency of the immune system is a cause of persistent, protracted inflammation, as is found in PO. One major contributor is diminished TLR4 expression in predialysis and HD patients, especially in subjects who are predisposed to infections [79]. Diminished TLR4 expression has been associated with reduced synthesis of TNF-α, IL-1β, IL-6, and IL-8 in response to LPS challenges [79]. In HD patients, it has been suggested that endotoxins contained in the dialysate might contribute to a decrease in TLR4 expression [80]. It is plausible that decreased activity or expression of TLR is a main factor for the dysfunction of antigen-presenting cells and predisposition to infection of these patients [79].

**Figure 1:**
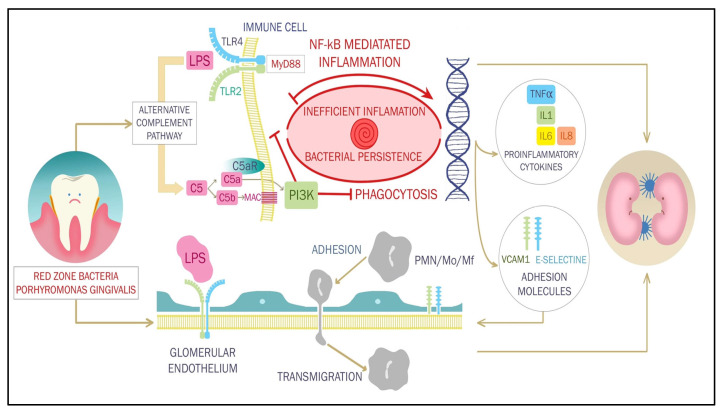
Toll-like receptor (TLR)2 and 4 mediated activation of innate immunity triggered by bacterial lipopolysaccharide of Porphyromonas gingivalis and other red zone bacteria leads to immune cell recruitment and activation both in periodontal tissues and the kidney. Nuclear factor k B (NF-kB)-mediated expression of proinflammatory cytokines like interleukin (IL)1 and 6, tumor necrosis factor (TNF)α, and adhesion molecules such as vascular cell adhesion molecule 1 (VCAM1) and E-selectins mediate polynuclear adhesion and transmigration in in various tissues, including the kidney. However, alternative intracellular cascades to the classical myeloid differentiation primary response gene 88 (MyD88) dependent pathway, phosphatidyl inositol 3 kinase (PI3K), or complement factor 5 (C5), cyclic AMP (cAMP), and protein-kinase-mediated membrane attack complex (MAC) activation-block normal phagolysosome activation of macrophages and neutrophils, leading to inefficient, protracted periodontal inflammation.

Pathogenic mechanisms linking PO to CKD are depicted in Figure 1.

### 3.3. Systemic Inflammation and Proinflammatory Cytokines in CKD and PO

CKD is a proinflammatory state characterized by an increase of inflammatory markers, including cytokines. Multiple lines of evidence have supported a direct pathogenic role for inflammation in progression of CKD, but also in development of various complications such as malnutrition, coronary artery calcification, atherosclerosis, cardiovascular disease, and enhanced CKD mortality. Relevant proinflammatory molecules that are increased in CKD are interleukin (IL) 6, TNFα, and adhesion molecules and adipokines [81,82]. A state of systemic inflammation, such as the one associated with CKD, can trigger PO, which was proven in transversal [83] and longitudinal studies [84]. In a recent scoping review, the relationship of systemic low-grade inflammation found in several systemic diseases (such as diabetes, obesity, and cardiovascular disease) to PO was explored; systemic proinflammatory cytokine levels such as interleukin IL1 and 6, tumor necrosis factor (TNF)α, and adhesion molecules were linked to prevalence and severity of PO [85]; CKD was not explored in this review. Nevertheless, several studies have reported increased levels of proinflammatory cytokines such as TNFα, IL6 and Il1 in CKD patients with PO [86]. Systemic increase in proinflammatory cytokines is paralleled by oral changes: cytokines in crevicular fluid such as TNFα and IL 8, as well as clinical indicators of the severity of PO such as plaque index, gingival index, and probing pocket depth or CAL are significantly increased in HD patients when compared to controls [87].

PO is also a cause of subclinical inflammation and associated with an increase of markers of systemic inflammation [88]. In their recent work, Mahendra [73] et al. found that the presence of periodontogenic bacteria strongly correlates to serum levels of TNF α and predicts a degree of kidney damage, as reflected by GFR kidney damage and of PO, such as plaque index, gingival index, probing pocket depth, or CAL. The cytokines reported as increased in PO patients with CKD notably TNFα, and proinflammatory cytokines are known to be involved in kidney damage in different models: IL 8 is involved in pathogenesis of glomerulonephritis [89], IL 17 is associated with kidney damage from hypertension [90], and TNFα participates in disease progression and contributes to renal inflammatory response in glomerular diseases such as diabetic nephropathy [91].

In CKD patients, the association of PO with increased inflammatory markers, such as C- reactive protein (CRP), has repeatedly been reported; in a recent systematic review this issue was studied. With 2 exceptions out of 8 reported studies, CRP was significantly elevated in CKD patients with PO [91]. Low albumin as a negative acute phase protein was also reported to be associated with PO [91]; however, serum albumin is also an important nutritional marker. The association of systemic inflammation to protein wasting and accelerated atherogenesis, the so-called malnutrition–inflammation–atherosclerosis syndrome, was first described some 20 years ago [92], and is an important contributor to atherogenesis, cardiovascular morbidity, and mortality in CKD patients.

One special mention is needed regarding the contribution of matrix metalloproteinases (MMP) in pathogenesis of PO- and CKD-induced lesions. MMPs are a group of enzymes involved in tissue repair and apoptosis, and some of them are upregulated during periodontal inflammation [93]. In the kidneys MMPs are involved in regulation of inflammatory response, but also in chronic fibrosis and progression of CKD; thus, PO-induced systemic overexpression of MMPs might contribute to kidney damage.

### 3.4. Endothelial Dysfunction and Oxidative Stress

Endothelium is much more than a mechanical barrier separating blood from tissues; it exerts active regulatory functions on the balance of oxidative stress, vasoconstriction/vasodilation, platelet aggregation, and leukocyte adhesion. PO can influence all these functions and, given the fact that kidneys receive up to 25% of cardiac output to assure glomerular filtration, the effects of PO on glomerular endothelial function are significant [94,95].

Porphyromonas gingivalis LPS has been detected in various endothelial cells; it triggers induction of increased reactive oxygen species (ROS), followed by NF-kB-induced inflammation, polynuclear adhesion, and cell apoptosis. ROS produced by leukocytes during inflammation are an important defense mechanism in PO targeting bacterial DNA [96], however excessive inflammation, triggered by the escape mechanisms described above, generates excessive ROS, which leads to systemic imbalance between prooxidative and antioxidant species with potential effects on various organs, including the kidney. Indeed, increased oxidative stress is an important characteristic of CKD, related to microinflammatory environment in these patients, and detected early in the course of the disease, tending to worsen with time [97]. One contributor to increased oxidative stress might be PO. Human studies suggest that some markers of systemic oxidative stress such as serum 4-hydroxy-2-nonenal (4-HNE) are associated to severity of PO [98]. In another study, glutathione-marker of oxidative stress was significantly higher (and related to periodontal parameters) in patients with CKD and PO than in patients with CKD only [30].

As mentioned before, an analysis of a large longitudinal cohort seems to indicate that CKD and PO are reciprocal risk factors, and that one important mechanism for their interaction is oxidative stress [49].

PO also influences vasodilation/vasoconstriction balance; endothelial dysfunction measured by flow-mediated vasodilation of the brachial artery is associated with PO and various markers of inflammation [99].

Oxidative stress is frequently observed in CKD and dialysis patients and is nowadays considered a non-traditional risk factor for all-cause mortality in CK [100,101]. Elevated levels of ROS are mainly due to the impairment of their physiological defense mechanisms and to addition of nitrogen containing oxidative species [82,102]. At the renal level, oxidative stress is responsible for progressive renal damage, *glomerulosclerosis*, and interstitial fibrosis, exacerbating the severe inflammatory processes already underway. At a systemic level, CKD-related oxidative stress is also responsible for several pathological conditions contributing to morbidity and mortality such as atherosclerosis and cardiovascular disease. Last, but not least, ROS are able to further enhance the inflammatory response by triggering pro-inflammatory mediators (e.g., NF-κB related proinflammatory cascade) [82].

### 3.5. Metabolic and Homeostatic Changes in CKD and Their Influences on PO

**Figure 2:**
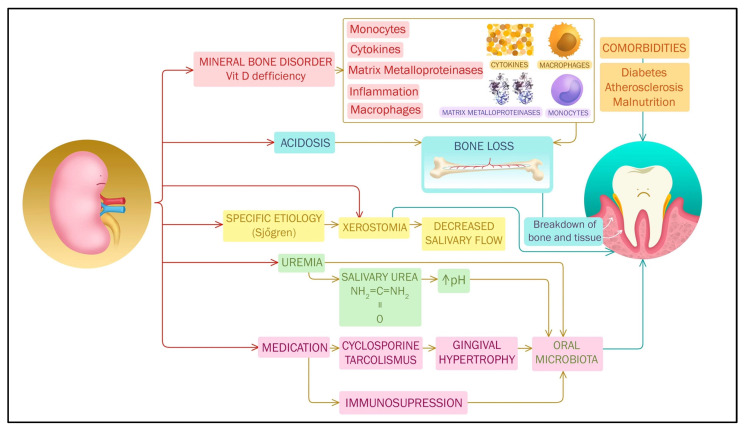
Homeostatic imbalances caused by CKD may be involved in pathogenesis of PO in various ways: vitamin D deficiency may impair immune response and conjugated with other mineral bone disorders and acidosis may contribute to periodontal bone loss; xerostomia in advanced CKD, especially dialysis patients and/or due to specific etiologies such as Sjogren syndrome, impairs salivary flow; uremic toxins, increased salivary pH, and specific medication, especially immunosuppressants, cause dysbiosis; comorbidities of CKD may independently influence PO.

Alterations to internal homeostasis of CKD that might be relevant for pathogenesis of PO are depicted in Figure 2.

The term ‘mineral bone disorders’ depicts the whole spectrum of abnormalities of biochemical parameters, bone structure changes, and vascular calcifications secondary to altered mineral metabolism of CKD. Among biochemical parameters, hypocalcemia, hyperphosphatemia, increased fibroblast growth factor 23 (FGF23), parathormone (PTH), and alkaline phosphatase, as well as 1,25-dihydroxy vitamin D3 deficiency, are the most common findings. It is not clear to what extent mineral bone disorders of CKD might be linked to PO. One study identified positive correlation of serum calcium and phosphate to the severity of PO reflected by CAL (but, noteworthily, not by other periodontal parameters, such as pocket depth, gingival index, plaque index, or bleeding on probing) [103]. However, these findings are not universal, and serum calcium was found in one report as a protective factor regarding PO [104]. Other authors found similar [52] or even lower phosphate levels in hemodialysis patients with PO. Similarly, PTH was not found to be correlated to PO [104] or alveolar bone loss [105] in dialysis patients; nor were total serum alkaline phosphatase levels [29,52]. However, in CKD patients, crevicular fluid alkaline phosphatase was higher in PO sites than in healthy ones, and in sites with mere gingival hypertrophy and three times higher than the serum levels, indicating that periodontal bone might be a source of alkali phosphatase [106]. Higher levels were associated with clinical parameters of PO.

In a small case-control study, vitamin D deficiency was more severe in patients with CKD and PO than in controls without PO [107]; clinical parameters of severity of periodontal disease were also related to vitamin D levels in a cross-sectional study [108]. In populational cohorts, vitamin D levels are associated with periodontal tooth loss. This association might be explained by the pleiotropic effects of vitamin D, including anti-inflammatory action involving reduction of Il 17, TNF alfa, and IL 6 through the inhibition of NF-kB [109,110]. In fact, vitamin D deficiency is associated with higher salivary levels of putative proinflammatory cytokines, such as Il 17 and MMP 9, in PO patients. One other possible antimicrobial effect of vitamin D is mediated via stimulation of macrophages and monocytes to produce proteases–beta defensins [109] with antibacterial effects [111] following the binding to the vitamin D receptor. In fact, certain single nucleotide polymorphisms of vitamin D receptor have been associated with PO [112]. Another putative mechanism is promotion of autophagy of cells with internalized live of Porphyromonas gingivalis [113]. Additionally, vitamin D deficiency can influence alveolar bone healing. Vitamin D supplementation seems to be a crucial regulator of immune homeostasis and inflammatory regulation in rats [114]. Its effect upon the decrease of salivary inflammatory cytokines has been studied, but clinical relevance is not certain [115]. Although a small study suggests the benefit of vitamin D supplementation on PO [116], a recent randomized controlled trial did not confirm these results [117].

In a mouse model of diabetic nephropathy Porphyromonas gingivalis, LPS exposure induced accumulation of FGF 23 in kidneys [118]. FGF 23 is a putative factor for ventricular hypertrophy and hypertension and might contribute to the cardiovascular mortality of these patients.

Metabolic acidosis is yet another homeostatic imbalance associated to renal disease: usually high anion gap acidosis, due to accumulation of phosphate and sulphate containing acids, is found, but hyperchloremic acidosis due to tubular dysfunction might also contribute; significant acidosis becomes prevalent in stage 4 CKD and is constant in dialysis patients. Long-standing acidosis is known to cause bone loss and might contribute via decreased support of alveolar bone to PO [119].

Saliva is a major controlling factor of intraoral biofilm; decreased salivary flow and decreased oral hydration can cause both gingivitis and PO. Xerostomia is an important symptom of CKD; it might be present in pre-dialysis phases in certain conditions, e.g., rheumatic disease such as Sjogren syndrome. Singh et al. demonstrated that, in Sjogren syndrome patients, the number of sites with CAL ≤ 4 mm was significantly higher compared to the healthy controls [120]. Decreased salivary flow is a major issue in dialysis patients, in whom renal output is decreased and water intake is hence restricted; mouth breathing might also be a contributor [121].

Increased salivary urea, whose hydrolysis engenders an alkaline salivary pH [72], can influence periodontal pathogen proliferation and calculus deposition. Interestingly, this alkaline pH might be responsible for less cavities observed in CKD patients [122].

For unclear reasons, in end-stage renal disease patients, dialysis modality might play a role with HD, having a negative impact on oral health and on indices like visible plaque index, CAL, and candida colonization. The effect is reversible when switching to PD [123].

## 4. Confounding Disease

### 4.1. Diabetic Nephropathy

Association of PO with other comorbidities of CKD should be considered when analyzing a possible pathophysiologic link between these conditions. Extensive evidence suggests that diabetes is more prevalent in PO patients, with these facts confirmed in a recent systematic review [124]. Diabetic nephropathy is the leading cause of CKD in developed countries, accounting for nearly 50% of end-stage renal disease cases, which might account in part for the increased prevalence of PO in CKD. Patients with diabetes and nephropathy have a higher risk of having missing teeth than those without nephropathy; moreover, PO is more severe in advanced dialysis dependent diabetic patients than in non-dialysis ones [125]. Noteworthily, PO is also associated with the progression of diabetic nephropathy [45].

### 4.2. Cardiovascular Disease

As noted above, cardiovascular disease and associated mortality are major problems in CKD, both in predialysis and in dialysis stages. PO is recognized as a major contributor to atherosclerosis via endothelial disfunction, inflammation, and progression of atherosclerotic plaques [95,126]. It is not surprising that PO is independently associated with cardiovascular morbimortality of CKD [4,51,127].

### 4.3. Effect of Medication

Immunosuppressive treatment (e.g., that of glomerular disease or kidney transplantation) can also contribute to propagation of PO. The bacterial species associated with PO in immunosuppressed patients depends on the immunosuppression regimen, with certain species (Pavimonas micra and Capnocytophaga species) favored by corticoid and mycophenolic acid salt therapy. Worse periodontal condition is described in patients under cyclosporine when compared to other regimens [128]. However, even if there is an increased prevalence of pathogen bacteria characteristics of PO in immunosuppressed transplanted patients, the clinical impact is not clear.

A special mention needs gingival overgrowth, a common side-effect of calcineurin inhibitors used for treatment of various glomerular conditions and for kidney transplantation. In the latter setting, up to 10% of patients have gingival hypertrophy [129]. Tacrolimus produces slightly less overgrowth than cyclosporin and it occurs later in time [130], but all patients under calcineurin inhibitor therapy should receive special attention.

## 5. Treatment

Treatment of CKD is standardized; a part of the etiologic treatment, it is addressed for halting progression of CKD, treating complications and comorbidities, and replacing renal function by dialysis or transplantation. We have found no prospective studies or trials on the impact of a specific CKD treatment on PO as an outcome.

Standard therapy in PO is outlined in the current treatment guidelines [131] and consists of (a) improving behavioural practices, supragingival biofilm, gingival inflammation and risk factor control; (b) supra- and sub-gingival instrumentation, with and without adjunctive therapies; (c) different types of periodontal surgical interventions; and (d) the necessary supportive periodontal care to extend benefits over time. However, there are currently no globally implemented guidelines for the treatment of PO in CKD patients, as there is a paucity of studies addressing this issue.

Among prophylactic measures, oral hygiene is important, from the perspective of it being correlated to gingival overgrowth [132]. Improved oral hygiene was associated with decreased occurrence of CKD in a nationwide retrospective cohort study [133]; likewise, frequency of brushing had a positive impact on GFR decline or need of dialysis in a large retrospective study [134]. Prophylaxis of PO was also assessed in a CKD in the pediatric population: one pilot study conducted on 30 patients aged 6–26 years compared intensive prophylaxis and treatment as usual (done on a control group of 15). No significant difference in CRP or prevalence of bacteremia was found; nevertheless, there was an important decrease of 90% of the gingival index (GI) in the intensive dental prophylaxis group that led to an almost healthy gingiva fact, showing that there is a chance to reduce systemic inflammation in CKD pediatric patients [135].

Regarding treatment of PO, there is indirect evidence of the potential benefit of non-surgical treatment on inflammatory markers: intensive treatment of PO was found to be associated to decrease of markers of inflammation and endothelial dysfunction [136] [91,98,137] and to improvement of nutrition parameters and iron availability in dialysis patients on peritoneal and/or HD [31,138,139].

There are few prospective studies with endpoints defined according to renal or PO outcomes in these patients at risk. Non-surgical treatment of PO lead to improved renal function in small prospective studies [134,136,140,141], with findings confirmed by a nationwide cohort study from China that found decreased progression to end-stage renal disease in CKD patients treated for PO [142]. There is a paucity of randomized controlled trials regarding the effect of PO treatment on renal outcomes. Non-invasive nonsurgical intensive treatment was proven to improve dental prognostic in CKD patients with PO [141,143]. Concerning systematic review and meta-analysis, there are at least three recent attempts to analyze data from available evidence, which included largely different studies and reached different conclusions [144,145]. The most recent and complete one suggested a positive effect of PO treatment on GFR in CKD patients [146].

Treatment of PO in CKD patients may be more difficult, as pathogenic subgingival microbiota tends to persist after periodontal treatment [147].

Given the vast evidence of association of PO to CKD, one should expect increased awareness of this matter in both renal physicians and dentists in order to generate evaluation protocols and treatment guidelines. However in this matter there is room for improvement: most renal physician and nurses, despite having correct knowledge on PO, do not routinely inspect oral cavities of patients, and they refer them to dental care in less than 30% of cases [148]. Additionally, due to general health status and comorbidities, dental visits in patients with CKD are less likely; these results form a retrospective analysis of US public dental health-care system records [149]. Additionally, self-awareness of the patients regarding PO is low, and patients are less likely to receive adequate dental care [150].

PO is not listed as a complication for CKD and not addressed by current KDIGO guidelines. Additionally, current PO guidelines [131] do not require assessing presence CKD in PO patients. Future prospective trials should ascertain if protocolized evaluation and standardized intervention can improve outcomes for these patients.

## 6. Strengths and Limitations

Our review aims to present data on the interplay of PO and CKD, a necessary step given the overwhelming evidence of epidemiologic association between the two conditions and their respective impact on general health. The strength of our review is the complex perspective from both nephrologists’ and dentists’ point of view regarding pathogenetic pathways that can play a role in the bidirectional relationship between the two conditions. Understanding mechanisms of oral and systemic inflammation and homeostatic imbalances in PO patients with CKD is key to efficient intervention. The limitations of our work are the inability to draw conclusions on best clinical practice in PO patients with CKD due to insufficient data derived from clinical trials or prospective studies, regarding cost-effectiveness and outcomes of specific therapeutic measures. This holds true when referring to the lack of data on optimal evaluation of these patients.

## 7. Conclusions

There is overwhelming evidence of the association of CKD and PO, however a few studies also support directional relation between the two pathologies. PO and CKD share common pathogenic mechanisms such as protracted, inefficient inflammation, oxidative stress, and endothelial dysfunction; PO may be facilitated by homeostatic changes found in CKD, comorbidities, or immunosuppressive treatment. Further prospective studies as well as randomized controlled trails, especially clarifying effects of PO treatment on renal outcomes, are needed for better insight into the relationship between CKD and PO.

## Data Availability

Not applicable.
